# The Effect of PEGylated Graphene Oxide Nanoparticles on the Th17-Polarization of Activated T Helpers

**DOI:** 10.3390/ma16020877

**Published:** 2023-01-16

**Authors:** Svetlana Zamorina, Valeria Timganova, Maria Bochkova, Kseniya Shardina, Sofya Uzhviyuk, Pavel Khramtsov, Darya Usanina, Mikhail Rayev

**Affiliations:** 1 Branch of the Perm Federal Research Center, Ural Branch of the Russian Academy of Sciences, Institute of Ecology and Genetics of Microorganisms, Goleva st., 13, Perm 614081, Russia; 2 Department of Microbiology and Immunology, Faculty of Biology, Perm State National Research University, Bukireva st., 15, Perm 614990, Russia

**Keywords:** PEGylated graphene oxide, cytokine profile, IL-17-producing helper T cells (Th17), naïve T helper polarization

## Abstract

We investigated the direct effect of PEGylated graphene oxide (P-GO) nanoparticles on the differentiation, viability, and cytokine profile of activated T helper type 17 (Th17) in vitro. The subject of the study were cultures of “naive” T-helpers (CD4+) isolated by immunomagnetic separation and polarized into the Th17 phenotype with a TCR activator and cytokines. It was found that P-GO at low concentrations (5 µg/mL) had no effect on the parameters studied. The presence of high concentrations of P-GO in T-helper cultures (25 μg/mL) did not affect the number and viability of these cells. However, the percentage of proliferating T-helpers in these cultures was reduced. GO nanoparticles modified with linear polyethylene glycol (PEG) significantly increased the percentage of Th17/22 cells in cultures of Th17-polarized T helpers and the production of IFN-γ, whereas those modified with branched PEG suppressed the synthesis of IL-17. Thus, a low concentration of PEGylated GO nanoparticles (5 μg/mL), in contrast to a concentration of 25 μg/mL, has no effect on the Th17-polarization of T helpers, allowing their further use for in-depth studies of the functions of T lymphocytes and other immune cells. Overall, we have studied for the first time the direct effect of P-GO nanoparticles on the conversion of T helper cells to the Th17 phenotype.

## 1. Introduction

Graphene is a two-dimensional allotropic modification of carbon. It has a number of unique properties, such as the ability to fluoresce, catalytic and antimicrobial activity, and a high specific surface area. The combination of these properties makes it possible to consider the use of graphene-based materials in the field of biomedicine, especially for targeted drug delivery, such as adjuvants, etc. The introduction of graphene-based drugs is complicated by the cytotoxicity of graphene and its pro-inflammatory effects [1,2]. The situation is complicated by the fact that the nature of graphene’s effect on cells is determined by many factors such as particle size, configuration, concentration, etc. [3]. However, with the proper selection of these properties, a significant reduction in the cytotoxic effect can be achieved. It is also known that some negative effects are leveled when the surface of graphene nanoparticles is functionalized with biocompatible polymers: The stability of graphene under physiological conditions is increased, the interaction with other biomolecules is minimized, and the risk of immune reaction is reduced [4,5,6]. For this reason, graphene oxide (GO) is more commonly used than graphene in work, as there are carboxyl groups on its surface that facilitate the modification process. Substances such as gelatin, polyethylene amine (PEA), amino groups, polyvinylpyrrolidone (PVP), polyethylene glycol (PEG), etc. are used for functionalization.

Given the many possibilities for the use of graphene-based drugs, a comprehensive investigation and evaluation of the biocompatibility of graphene, especially its interaction with cells of the immune system, since these cells are primarily exposed to nanoparticles, becomes an urgent task. Currently, there are data on the effect of graphene oxide on different types of cells of the immune system, which are often contradictory. For example, graphene oxide nanoparticles are known to damage the membrane and increase the production of reactive oxygen species (ROS) by neutrophils [7], but in contrast, ROS production by monocytes decreases [8]. However, the effect of GO nanoparticles on the immune system is not limited to the influence on innate immune cells. It is known that unmodified GO suppresses the proliferation of T lymphocytes [7] and reduces the viability of activated T helpers [3]. There is evidence for a dendritic cell-mediated effect of graphene quantum dots on T cell response [9]. However, the direct effects of GO on the differentiation and polarization of T helpers have not yet been investigated.

T helpers (CD4+ T cells) are important participants in the immune response and can regulate it in different directions due to their ability to polarize into many subpopulations. Currently, the following major subpopulations of T helpers are known and are listed as follows: Th1, Th2, Treg, Th9, Tfh, Th17, Th22. Th17 and Th22 cells carry the CCR6 molecule on the membrane, which determines their migration to sites of inflammation [10]. A common feature of these subpopulations is their involvement in both “normal” immune responses (combating fungi and extracellular bacteria, maintaining gut microbiota composition, mucosal and epidermal integrity) and pathological ones (autoimmune diseases, pregnancy complications, transplant rejection) [11,12,13,14,15]. In addition, there is evidence that Th17 and Th22 are involved in the pathogenesis of some cancers [16,17]. This Th17/Th22 duality is largely due to their high plasticity, i.e., the ability to transdifferentiate into Th1- and Treg-like cells in the presence of a proinflammatory or anti-inflammatory cytokine microenvironment, respectively [18].

Th1-like cells or Th17.1 produce IFNɣ in addition to the major Th17 cytokine IL17 and have been implicated in the pathogenesis of autoimmune diseases such as rheumatoid arthritis and multiple sclerosis according to some data [19,20].

Therefore, considering the important role of Th17 and Th22 in protective immunity, as well as their increased plasticity and ability to transform into pathological Th17.1, the aim of our work was to investigate the direct effect of PEGylated GO nanoparticles on Th17 polarization of T-helpers, in the context of the perspectives of the application of this material in biomedicine.

## 2. Materials and Methods

**Donors**. We used CD4+ cells isolated from peripheral blood mononuclear cells (PBMCs) from healthy female donors (*n* = 5). The study was conducted in accordance with the WMA Declaration of Helsinki 2000 and the Protocol of the Council of Europe Convention on Human Rights and Biomedicine 1999; approval of the Ethics Committee of the IEGM Ural Branch of the Russian Academy of Sciences (IRB00010009) dated 30 August 2019 was obtained for the experimental scheme used. Written informed consent was obtained from all patients. The authors adhered to all relevant ethical standards.

**Figure 1:**
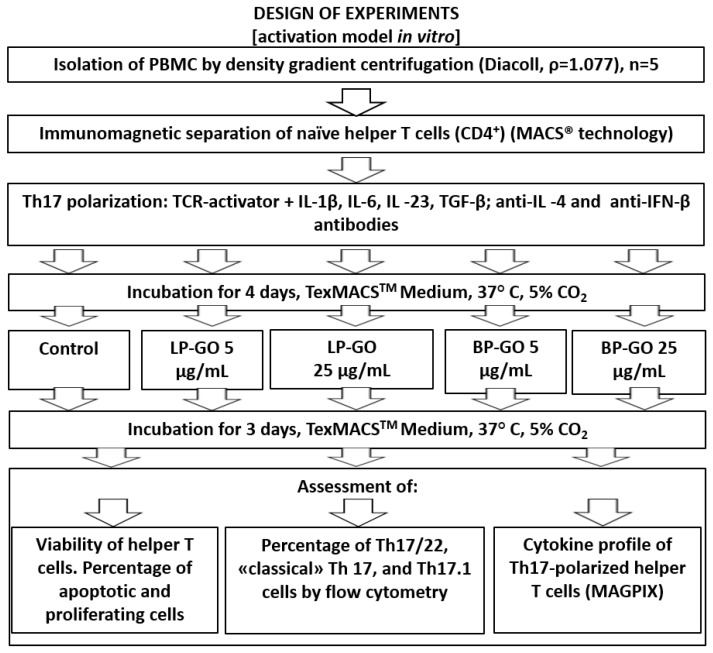
Experimental design.

**Study Design.** To evaluate the effects of graphene oxide nanoparticles on the differentiation, viability, and production of cytokines by T helpers, an activation model was used (Figure 1) that mimics the process of interaction of T lymphocytes with antigen-presenting cells (APCs) in the presence of pro-inflammatory cytokines (IL1β + IL6) [21]. The interaction of T-helpers with TCR activator, which replaces APC in vitro, triggers processes such as activation, differentiation, and production of cytokines.

**Figure 2:**
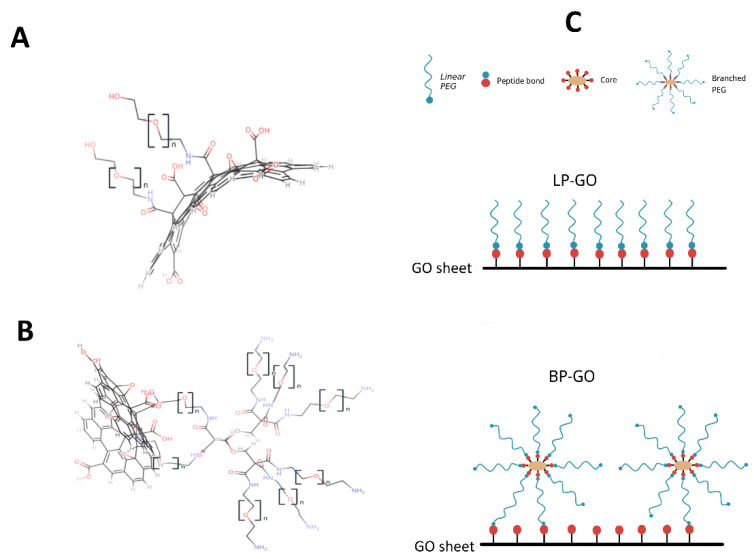
Structural formulas and a schematic representation of the surface of pegylated GO nanoparticles. (**A**) structural formula of LP-GO nanosheet, (**B**) structural formula of BP-GO nanosheet, (**C**) a schematic representation of both types of nanoparticles. Note: the ratio of the sizes of PEG and GO is not met.

**Graphene oxide.** Graphene oxide nanoparticles with a lateral size of 100–200 nm (Ossila Ltd., Sheffield, UK) were coated with linear (LP-GO) and branched (BP-GO) polyethylene glycol. Modification of GO was carried out by covalent bonding of the amino groups of PEG-NH_2_ and 8arm-PEG-NH_2_ to the surface carboxyl groups of GO. The modification procedure and characterization of these nanoparticles were described in our recent article [22]. The properties of the nanoparticles are summarized in Table 1. Structural formulas and a schematic representation of the surface of pegylated GO are shown in Figure 2.

**Detection of endotoxin contamination.** Endotoxin contamination of nanoparticles was analyzed using the Thermo Scientific™ (Waltham, MA, USA) Pierce™ LAL Chromogenic Endotoxin Quantitation Kit according to the manufacturer’s instructions. Prior to analysis, the tested concentrations of P-GO nanoparticles were diluted 50-fold with endotoxin-free water.

Isolation of CD4 cells. Peripheral blood mononuclear cells (PBMCs) were obtained by centrifugation in a Diacoll density gradient (1.077 g/cm^3^) (Dia-M, Moscow, Russia). To obtain naive CD4+ T cells from the PBMC suspension, the negative immunomagnetic separation method (MACS^®^ MicroBeads and MS Columns, Miltenyi Biotec, Bergisch Gladbach, Germany) was used. After separation, the number of cells and their viability were determined using Trypan Blue 0.4% (InVitrogen, Waltham, MA, USA). The purity of the isolated naive T cells was confirmed by staining CD45R0, CD45RA, and CD62L (CD45RA-FITC, CD45R0- PE (BioLegend, San Diego, CA, USA) and CD62L-APC (Miltenyi Biotec, Germany) on a CytoFLEX S flow cytometer (Beckman Coulter, Brea, CA, USA). The percentage of naive T cells (CD45R0-CD45RA+ CD62L+) was ~70% of the lymphocyte gate.

Culturing CD4+ cells. Isolated naive CD4+ cells were cultured in 96-well plates (concentration 10^6^ cells/mL, volume 200 µL) in serum-free complete culture medium (TexMACS™ medium (Miltenyi Biotec)) supplemented with 10 mM HEPES, 2 mM L-glutamine (ICN Pharmaceuticals, Costa Mesa, CA, USA) and penicillin-streptomycin-amphotericin B (BI, Bi’ina, Israel) in a humidified atmosphere in a CO_2_ incubator at 37 °C and 5% CO_2_ for 7 days without changing the medium.

To polarize lymphocytes into Th17 phenotype, recombinant cytokines IL-1β (20 ng/mL), IL-6 (30 ng/mL), IL-23 (30 ng/mL), TGF-β (2.25 ng/mL); anti-IL-4 antibody (2.5 µg/mL) and anti-IFN-β antibody (1 µg/mL), and TCR activator (MACSiBead^TM^ particles loaded with antibodies against human CD2, CD3, and CD28) were added to the cultures according to Miltenyi Biotec recommendations. LP-GO and BP-GO nanoparticles were added to the cultures on day 3 at final concentrations of 5 and 25 µg/mL. Cultures without GO nanoparticles served as controls. After the end of incubation, culture supernatants were clarified by centrifugation at 14,000 g and frozen.

**Figure 3:**
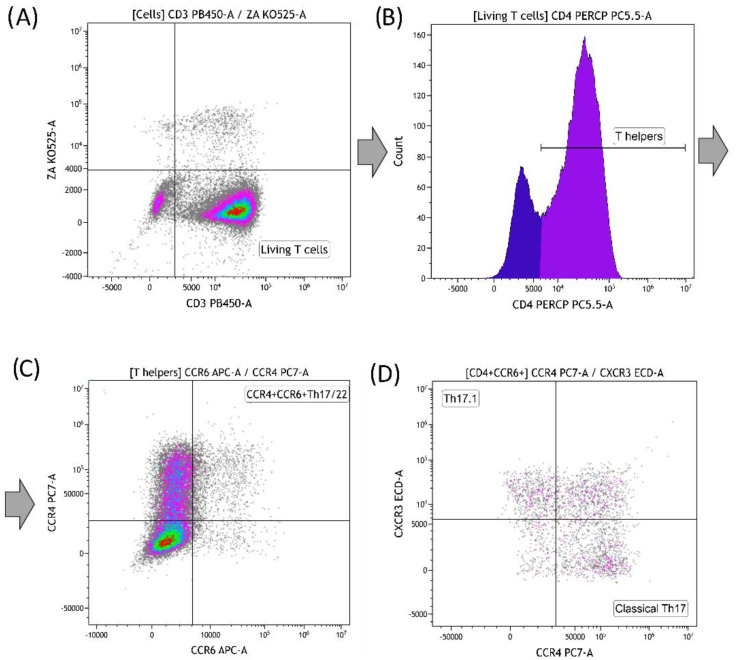
(**A**–**C**) Gating strategy of the Th17/22 subpopulation. ((**A**) live T cells; (**B**) T helpers; (**C**) Th17/22); (**D**) gating strategy of Th17 subpopulations (17.1 and “classical”).

**Flow cytometry.** After 7 days of cultivation, we determined the total percentage of live T lymphocytes (ZA-CD3+), the percentage of T helpers (ZA-CD3+CD4+) and their Th17/22 subpopulations (ZA-CD3+CD4+CCR4+CCR6+), and the percentage of CCR4+CXCR3- and CCR4-CXCR3+ cells (“classical” Th17 and Th17.1) in the CD4+CCR6+ population (Figure 3) according to Miltenyi Biotec recommendations and studies [21,23].

Sample preparation and surface staining were performed according to the antibody manufacturer’s instructions (Miltenyi Biotec, Bergisch Gladbach, Germany). Stained samples were analyzed using a CytoFLEX S flow cytometer (Beckman Coulter, Brea, CA, USA). Antibodies used are listed as follows: mouse IgG1 against human CXCR3-PE-Vio615 (REA 232 clone), CD4-PerCP (VIT4 clone), CCR4-PE-Vio 770 (REA279 clone), CCR6-APC (REA190) (all Miltenyi Biotec, Bergisch Gladbach, Germany), and CD3-Pacific Blue^TM^ (UCTH1 clone) (BioLegend, San Diego, CA, USA).

The threshold between positive (stained) and negative cell subpopulations was determined using unstained samples as well as fluorescence minus one (FMO) controls. Flow cytometry data were analyzed using Kaluza Analysis 2.0 software (Beckman Coulter, Brea, CA, USA).

**Figure 4:**
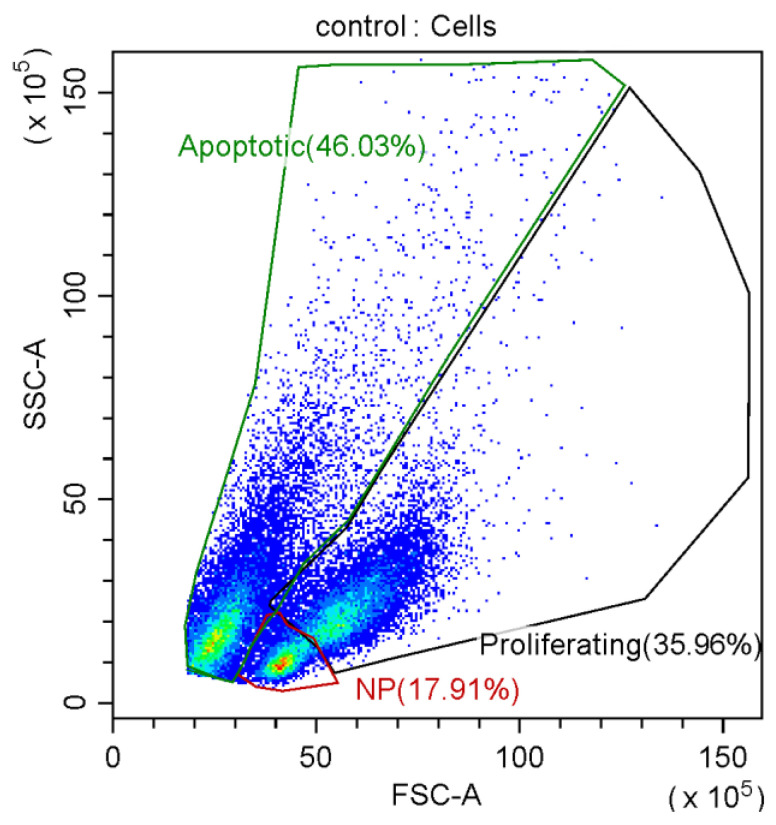
Example of differential gating dot plot. Note: NP—non-proliferating cells.

**Proliferation analysis.** The relative number of proliferating, non-proliferating, and apoptotic cells was determined using a modification of the differential gating method [24]. This method is based on changing the light scattering parameters of cells that are proliferating or apoptotic [25,26]. On the FSC/SSC plot, cells were first gated (cell debris was excluded), and then gates of non-proliferating, proliferating, and apoptotic cells were selected in the cell gate (Figure 4). Then, the percentage of cells falling into each of these gates was determined from the total number of cells [26,27]. Data were collected using a CytoFLEX S flow cytometer and analyzed using CytExpert software (Beckman Coulter, Brea, CA, USA).

**Cytokine profile assessment.** Subsequently, the concentrations of the following cytokines and chemokines in the supernatants were determined by multiplex analysis: IL-2, IL-4, IL-5, IL-7, IL-8, IL-10, IL-12 (p70), IL-13, IL-17, G-CSF, GM-CSF, IFN-γ, MCP-1, MIP-1β, TNF-α (Bio-Plex Pro^TM^ Human Cytokine Grp I Panel 17-Plex kit, Bio-Rad, Hercules, CA, USA). The assay was performed on a MAGPIX^®^ multiplex analyzer (Merck Millipore, Burlington, MA, USA) using xPONENT^®^ software. Standard curves were generated using a five-parameter logistic (5PL) analysis method. The data obtained were processed using Belysa^®^ Immunoassay Curve Fitting Software.

**Statistical data processing** was performed with GraphPad Prizm 8.0.1 software using the Friedman test and Dunn post hoc test for multiple comparisons. Results are presented as median, lower quartile, and upper quartile (Me (Q1–Q3)). The significance level was set at 0.05.

## 3. Results

### 3.1. Effects of P-GO on T Lymphocyte Viability

As a first step, the effect of graphene oxide nanoparticles on the viability of the total pool of lymphocytes (CD3+) was studied in vitro. The viability of cells in cultures with the addition of P-GO nanoparticles varied on average from 85.43% to 93.53%. No statistically significant effects of P-GO nanoparticles on the viability of these cells were observed compared to control samples (Table 2). This is consistent with previous data on other human immune cells from healthy donors. [22,28,29].

As for the effects of this material on T lymphocyte subpopulations, there is evidence from single-cell mass cytometry that GO nanoparticles at a concentration of 50 μg/mL had a cytotoxic effect on activated T helpers and cytotoxic T lymphocytes (CTLs), but at the same time this effect was significantly reduced when GONH_2_ was used [3]. GO and rGO at a concentration of 5 μg/mL had no effect on the viability of Th2 cells of the cell line SR.D10 [30]. It should be emphasized that cells in the above studies were exposed to GONH_2_/GO/rGO for a total of 24 h. In our experimental system, T helpers were first activated and then P-GO nanoparticles were added to the cultures and cultured for 72 h. Thus, we can say that 100–200 nm GO nanoparticles functionalized with linear and branched polyethylene glycol do not exhibit cytotoxicity against activated CD4+ T cells up to 25 µg/mL and up to 72 h of exposure. These data are important in terms of the prospects for the use of GO nanoparticles in biomedical research. However, even low concentrations of nanoparticles that do not affect the viability of cells may impair their functions upon long-term exposure to cells [31]. Therefore, it is important to study not only the cytotoxicity of nanoparticles, but also their effects on cell differentiation and function.

### 3.2. Effect of Graphene Oxide Nanoparticles on Proliferation of T-Helpers

**Figure 5:**
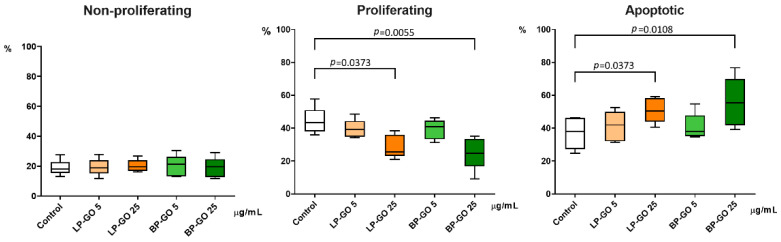
Percentages of non-proliferating, proliferating, and apoptotic cells in cultures of activated T-helpers with P-GO nanoparticles. Note: *n*= 5; the *x*-axis indicates the type and concentration of nanoparticles; the *y*-axis is the percentage of cells in corresponding gate from all cells. Control—culture without GO. Data are presented as median (Me) and quartiles (Q1–Q3).

The percentage of cells with light-scattering characteristics of proliferating cells was statistically significantly reduced in T-helper cultures with LP-GO and BP-GO at a concentration of 25 μg/mL compared to cultures without GO nanoparticles. Rate of cells with characteristics of apoptotic cells, on the contrary, was increased in these cultures (Figure 5).

In general, graphene-based materials can induce different types of programmed cell death [32]. The method we used is based only on cell morphology, but it is sufficient to see that the concentration of 25 µg/mL of GO nanoparticles we used causes a relative increase in the percentage of cells that have entered the path of cell death. We see that, unfortunately, functionalization with polyethylene glycol does not lead to abrogation of the apoptogenic effect of high concentrations of nanoparticles. However, it is quite possible to postulate that nanoparticles added at a lower concentration did not lead to critical changes in the proliferation status of cells.

### 3.3. Effect of Graphene Oxide Nanoparticles on Th17 Polarization of T-Helpers

Different subpopulations of T helpers can be determined by analysis of surface receptors for chemokines. The CCR6 receptor, whose ligand is the CCL20 molecule, is expressed on Th17 (IL-17a-producing T-helpers) and Th22 (IL-22-producing T-helpers) cells [10,14,21]. In general, the presence of the CCR6 molecule characterizes the T helper population, referred to as Th17/22 in our article, which is involved in the inflammatory response in autoimmune diseases and exhibits increased pathogenicity [33,34,35,36,37].

**Figure 6:**
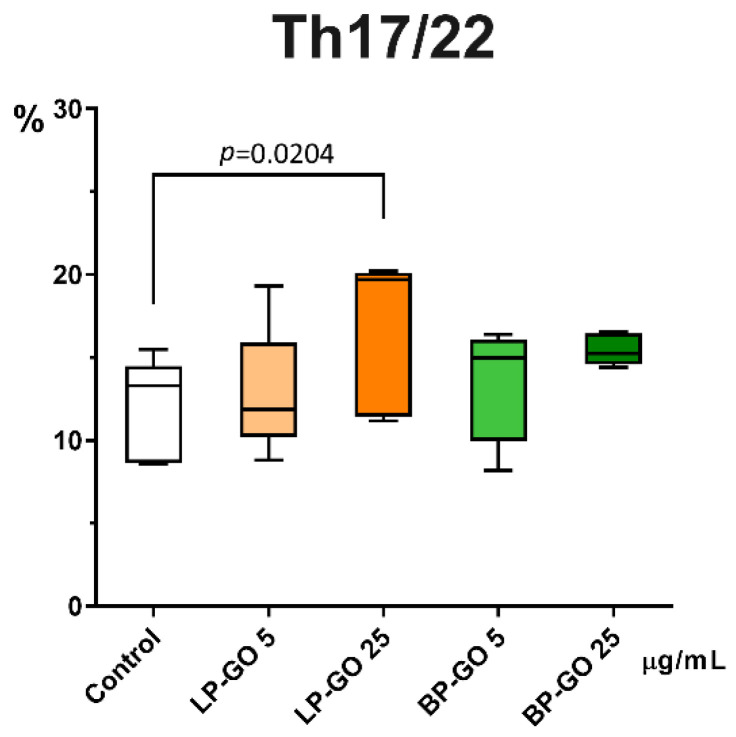
Percentage of Th17/22 cells (CCR4+CCR6+) from ZA-CD3+CD4+ lymphocytes in helper T cell cultures supplemented with pegylated GO particles in two concentrations. Note: the *x*-axis indicates the type and concentration of nanoparticles; the *y*-axis is the percentage of cells. Control—culture without GO. Data are presented as median (Me) and quartiles (Q1–Q3).

It was found that LP-GO nanoparticles at a concentration of 25 μg/mL significantly increased the percentage of Th17/22 cells in culture (Figure 6). BP-GO had no statistically significant effect on the percentage of Th17/22 cells.

CCR6+Th17 cells exhibit a considerable degree of plasticity of phenotype and function determined by cytokine environment and transcription factors [38]. Thus, several subpopulations of Th17 cells can be distinguished, depending on the expression of CCR4 and CXCR3 receptors and cytokines produced [39]. In our study, we analyzed the number of “classical” Th17 cells (CCR6+CCR4+CXCR3-) and Th1-like or Th17.1 cells (CCR6+CCR4-CXCR3+), which, as mentioned above, are considered to play an important role in the pathogenesis of inflammatory diseases of autoimmune nature.

**Figure 7:**
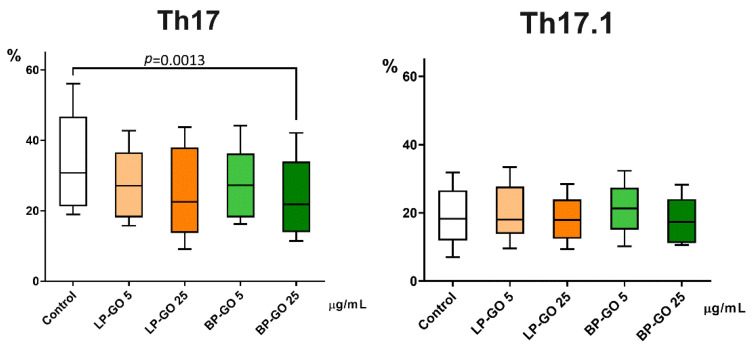
Percentage of “classical” Th17 (CCR4+CXCR3-) and Th17.1 (CCR4-CXCR3+) from CD4+CCR6+ cells in T helper cultures supplemented with PEGylated GO particles in two concentrations. Note: the *x*-axis indicates the type and concentration of nanoparticles; the *y*-axis is the percentage of cells. Control—culture without GO. Data are presented as median (Me) and quartiles (Q1–Q3).

LP-GO, which was added to cultures of polarized T-helpers, showed no effect, whereas BP-GO in high concentration caused a decrease in the number of “classical” Th17 cells. Neither type of nanoparticle had any effect on the proportion of Th17.1 cells (Figure 7).

Taking into account that in cultures with 25 μg/mL LP-GO the percentage of CCR6+CCR4+ T helper cells was increased and the percentage of “classical” Th17 cells did not change in this population, it can be assumed that the increase in the percentage of CCR6+CCR4+ cells is mainly due to Th22. These cells act similarly or even synergistically with Th17 in many cases, but have different surface molecules (CXCR10), transcription factors (AHR) and cytokines (e.g., FGF) [40]. Since the aim of our work was not to study this subpopulation, we can only speculate about this issue, so the above-mentioned effect of LP-GO should be studied in more detail.

### 3.4. Effect of Pegylated GO Nanoparticles on the Cytokine Profile of T Helpers Polarized into the Th17 Phenotype

**Figure 8:**
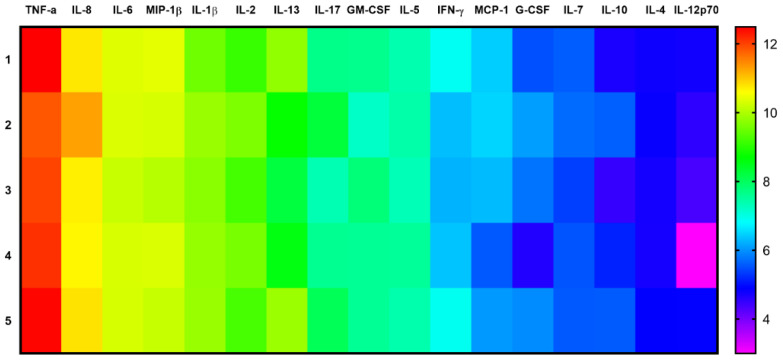
Heat map of cytokine concentrations in control cultures (without GO) of Th17-polarized helper T-cells. Note: *n* = 5; Cytokine concentrations are shown as natural log-values; IL-1β and IL-6 were introduced into the culture medium in accordance with Th17 polarization method.

In general, the chosen polarization scheme showed that the production of pro-inflammatory cytokines predominated in Th17-polarized helper T cell cultures without GO (Figure 8).

**Figure 9:**
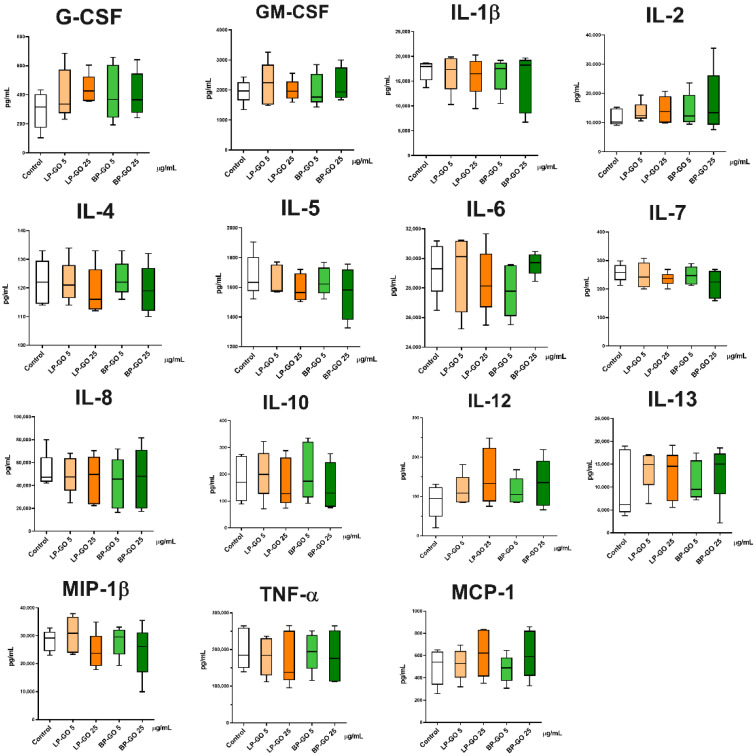
Cytokine concentrations in Th17-polarized cell culture supernatants after incubation with graphene oxide nanoparticles, *n* = 5, Me (Q1–Q3).

Cytokine profile analysis revealed that both types of PEGylated GO nanoparticles had no effect on the level of MIP-1β, MCP-1, IL-1β, IL-2, IL-4, IL-5, IL-6, IL-7, IL-8, IL-10, IL-12 (p70), IL-13, cytokines, and TNF-α, GM-CSF, G-CSF growth factors (Figure 9).

**Figure 10:**
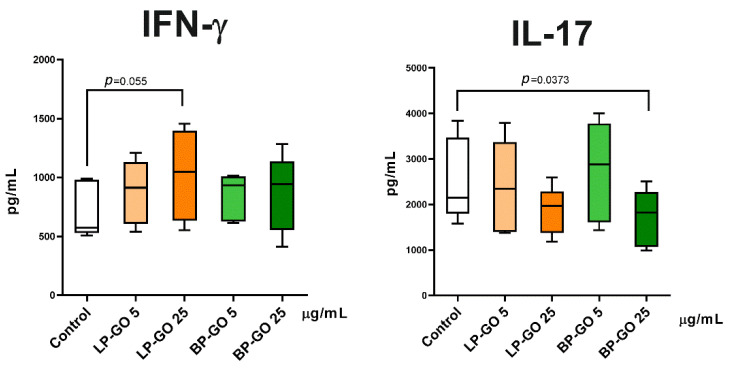
Production of IFN-γ (**left**) and IL-17 (**right**) by TCR-activated CD4+ cells polarized in the Th17 phenotype supplemented with pegylated GO particles at two concentrations. Note: *x*-axis indicates nanoparticle type and concentration of nanoparticles; the *y*-axis indicates cytokine concentration. Control—culture without GO. Data are expressed as median (Me) and quartiles (Q1–Q3). Significant differences (*p* < 0.05) compared with control are indicated.

However, it was found that LP-GO at a concentration of 25 μg/mL increased IFN-γ production (Figure 10).

Given that the proportion of CCR4-CXCR3+ cells (Th17.1), characterized by high IFN-γ production, in the Th17/22 population did not change during cultivation, it can be assumed that Th1 cells were the main source of this cytokine in this case. Not only innate immune cells, but also T lymphocytes express Toll-like receptors (TLR) [41,42,43,44,45]. At the present stage of research, it is known that not only specific ligands of microorganisms, but also other compounds, especially synthetic carbon compounds, acidic amino acids, and nanoparticles, can be responsible for the activation of these receptors [46,47]. In particular, the literature contains data on the ability of graphene oxide nanoparticles to trigger TLR -regulated inflammatory responses: In a study by Chen et al. [48], when mouse macrophages were cultured with GO (100 μg/mL), an increase in the secretion of IL-2, IL-10, IFN-g, and TNF-α was observed, mediated by the activation of TLR4 and TLR9. Lower concentrations of GO (50 μg/mL) also increased secretion of TLR-4, TLR-9, MyD88, and TRAF6 [49].

However, we should not forget that bacterial fragments (endotoxins) contaminating nanoparticles during their synthesis and/or use can cause research artifacts. After obtaining the data from this study, we decided to test the presence of endotoxin in suspensions of GO nanoparticles (Table 3). Since Th1 cells have been shown to respond directly to short-chain LPS by significantly increasing TLR4 expression and IFN-ɣ release [50], the presence of endotoxin in the suspension of LP-GO nanoparticles could explain the increase in IFN-ɣ in cultures of activated T helpers. However, TLR-4 lymphocyte activation has been shown to require soluble CD14 [50], which in turn is produced by activated monocytes [51]. However, the presence of monocytes in a culture where LPS is present and their subsequent activation should have caused an increase in the production of primary cytokines such as IL-1β and TNF-α [52], which we do not observe (Figure 9), so the issue remains open for discussion.

BP-GO nanoparticles at the concentration of 25 μg/mL decreased the production of IL-17, a key cytokine of Th17 cells, in cultures of activated T helpers (Figure 9). Because CCR4+CXCR3- cells, the so-called “classical” Th17, are characterized by high IL-17 and low IFN-γ production, these data confirm the inhibitory effect of BP-GO on the differentiation of classical Th17 in cultures. However, this effect cannot be described as pronounced. The percentage of “classical” Th17 decreased 1.4–1.5-fold, whereas the concentration of IL-17 decreased only 1.2-fold. By comparison, the combination of tacrolimus (1 ng/mL) and resveratrol (50 μmol) caused a two-fold decrease in the percentage of Th17 and a three-fold decrease in the expression of IL-17 mRNA [53].

## 4. Discussion

The general vector of effects of the P-GO nanoparticles we synthesized is consistent with those of our previous studies on other cells of the human immune system. We found that the particles at both concentrations used have no statistically significant cytotoxic effect on dendritic cells, monocytes, NK and NKT cells [29]. However, a tendency to decrease the percentage of live cells was still observed in cultures with a concentration of 25 μg/mL nanoparticles. Moreover, P-GO at high concentrations had an apoptogenic effect on NK cells. LP-GO caused an increase in the percentage of NK in early apoptosis, while BP-GO increased the percentage of cells in the stage of late apoptosis/necrosis. When we examined the protein corona of nanoparticles, we found that less protein was sorbed on particles coated with branched PEG than on particles coated with linear PEG. We hypothesized that the decrease in protein adsorption at the surface of BP-GO may lead to an increase in the apoptogenic properties of nanoparticles [29]. This assumption is supported by data showing that adsorption of proteins on the surface reduces the toxic effects of carbon nanotubes [54], and that protein corona attenuates the cytotoxicity of GO. Moreover, the adsorption of albumin on the GO surface actually weakens the interaction between phospholipids and the graphene surface, and significantly reduces the penetration of graphene and the damage to the lipid bilayer [55]. In the case of our BP-GO particles, it is likely that the denser PEG layer (due to its branched structure) and the higher molecular weight of 8arm-PEG determines the decrease in protein sorption on the particles and reduces the thickness of the protein corona [56].

Regarding the effects of GO specifically on T-helpers, a 2017 study using single-cell mass cytometry (CyTOF) found that pristine GO (50 μg/mL; particle size: 50 nm–1 μm) caused extensive nonspecific activation of T cells and stimulated the production of a large number of cytokines, in particular IL-2, IL-4 and IL-5 by cytotoxic lymphocytes and T-helpers. In contrast, GO nanoparticles functionalized with amino groups elicited a more specific response: Such nanoparticles induced the production of IL-2 and TNFα, but did not affect the synthesis of IL-5 [3]. In our study, GO nanoparticles functionalized with two types of PEG also did not induce a massive nonspecific cytokine response. Interestingly, in the above study, a greater effect of GO and GONH_2_ was observed on activated T cells than on naive T cells. The decrease in the production of IL-17 on addition of 25 μg/mL BP-GO to the cultures of activated T helpers is probably due to the fact that these particles showed their cytotoxic and/or apoptotic effect with respect to the most activated/proliferating population of “classical” Th17. The lowest percentage of proliferating cells and the highest percentage of apoptotic cells were found in these cultures, in agreement with the differential gating data (Figure 5). The lowest percentage of live cells was also found in cultures with 25 μg/mL of BP-GO (Table 2).

Epigenetic factors, such as post-translational histone modifications and DNA methylation, play a key role in the polarization and high plasticity of Th17 cells, manifested by the ability to transdifferentiate into Th1 or Treg, as well as in the expression of RORɣτ in association with GATA3 or FOXP3 [38]. Many effects of GO on various epigenetic processes have been described so far [57]. It is likely that the epigenetic toxicity of GO nanoparticles persists after their PEGylation and may affect Th17 polarization, a process that is highly dependent on epigenetic modifications.

In general, the results of the study highlight the importance of investigating not only the cytotoxicity of graphene oxide nanoparticles, modified with polymers, but also their epigenetic toxicity and their effects on proliferation and polarization, as the key stages of T helper differentiation.

The effect of reducing the proportion of “classical” Th17 in T-helper cultures was discovered for the first time and needs further research and unravelling of its mechanism.

## 5. Conclusions

This is the first time that data are available on the direct effect of graphene oxide nanoparticles on Th17 cells. It was found that P-GO had no significant effect on the viability of T lymphocytes in culture. However, both types of graphene oxide nanoparticles in high concentration caused a decrease in the percentage of proliferating cells in accordance with their light scattering properties. It was shown that LP-GO nanoparticles at high concentration increased the number of CCR6+Th17/22 cells and BP-GO at similar concentration reduced the percentage of “classical” Th17 in cultures of Th17-polarized T-helpers. This effect was accompanied by a decrease in the concentration of IL-17 in the culture supernatants.

A concentration of 5 µg/mL of P-GO nanoparticles does not affect Th17 polarization of activated T-helpers and does not affect the cytokine profile. Therefore, this concentration can be used in the future for in-depth studies of the functions of T lymphocytes and other immune cells.

## Figures and Tables

**Table 1:** Characteristics of the P-GO nanoparticles.

	LP-GO	BP-GO
Hydrodynamic diameter, nm ^1^	184 ± 73	287 ± 52
Polydispersity index	0.25 ± 0.02	0.23 ± 0.02
Zeta potential, mV	−31.70 ± 1.70	−34.28 ± 0.41
PEG mass fraction, %	17.2 ± 1.4	20.5 ± 1.8

^1^ measured by dynamic light scattering.

**Table 2:** Viability of lymphocytes in cultures with P-GO nanoparticles.

	Control	LP-GO 5 μg/mL	LP-GO 25 μg/mL	BP-GO 5 μg/mL	BP-GO 25 μg/mL
% of ZA-CD3+ cells	92.12 (88.61–94.22) *	93.53 (86.6–95.28)	91.12 (85.23–92.51)	92.58 (88.69–95.13)	85.43 (80.85–93.2)
*p* value	-	>0.9999	0.1112	>0.9999	0.182

* *n* = 5; Data are presented as median (Me) and quartiles (Q1–Q3). *p* value compared with the control are indicated.

**Table 3:** Concentration of endotoxin in P-GO nanoparticles, EU/mL (ng/mL).

	5 μg/mL	25 μg/mL
LP-GO	2.3 (0.23)	11.5 (1.15)
BP-GO	0.6 (0.06)	2.8 (0.28)

## Data Availability

The datasets used and/or analyzed during the current study are available from the corresponding author on request.

## Note for the Graphical Abstract

Parts of the figure were drawn by using pictures from Servier Medical Art. Servier Medical Art by Servier is licensed under a Creative Commons Attribution 3.0 Unported License (https://creativecommons.org/licenses/by/3.0/), accessed on 2 December 2022.
